# Non-Bosonic Damping of Spin Waves in van der Waals Ferromagnetic Monolayers

**DOI:** 10.3390/nano15100768

**Published:** 2025-05-20

**Authors:** Michael G. Cottam, Bushra Hussain

**Affiliations:** 1 Department of Physics and Astronomy, University of Western Ontario, London, ON N6A 3K7, Canada; 2 Department of Natural Sciences, University of Michigan, Dearborn, MI 48197, USA; bhussai@umich.edu

**Keywords:** spin waves, ferromagnetic monolayers, van der Waals ferromagnets, damping, renormalization effects

## Abstract

The spin wave renormalization processes in two-dimensional van der Waals ferromagnetic monolayers are investigated using an established non-bosonic diagram technique based on the drone-fermion perturbation method. The aim is to evaluate the damping of the long-wavelength spin wave modes at temperatures below the Curie temperature. In addition to the multi-magnon scattering processes, which typically dominate at low temperatures, an additional mechanism is found here that becomes important at elevated temperatures. This spin disorder damping mechanism, which was mainly studied previously in bulk magnetic materials and thicker films, features a spin wave or magnon being scattered by the magnetic disorder that is present when a longitudinal spin component undergoes large thermal fluctuations. The magnetic ordering in the monolayers is stabilized by an out-of-plane single-ion or Ising-type anisotropy, which influences the damping properties. Numerical results are derived for monolayer films of the van der Waals ferromagnet Cr_2_Ge_2_Te_6_.

## 1. Introduction

The renormalization of spin waves (or magnons) in ordered magnetic materials, such as ferromagnets and antiferromagnets below their critical temperatures, has long been a topic of intense interest, predominantly in bulk magnetic materials. Renormalization effects include the spin wave energy correction and damping as the temperature is increased (see, e.g., [1,2,3,4,5,6]). The spin wave damping (or reciprocal lifetime) has been studied especially in bulk-like magnetic materials and at low temperatures below the transition temperature. On the experimental side, the damping may be readily deduced as a contribution to the observed linewidths seen in techniques such as inelastic neutron scattering, Brillouin and/or Raman scattering of light, and magnetic resonance measurements [4,7,8,9,10,11,12,13].

Hamiltonian-based theories for the spin wave (SW) renormalization due to nonlinear processes at low temperatures frequently employ a transformation, such as the Holstein–Primakoff [14] or Dyson–Maleev transformations [15,16], to transform spin operators into boson operators (see, e.g., [1,2,3,4,5,17,18]). This approach is quite successful for sufficiently low temperatures that are typically below about half of the critical temperature, i.e., the Curie temperature $T_{C}$ for a ferromagnet and the Néel temperature $T_{N}$ for an antiferromagnet. At more elevated temperatures, high-density diagrammatic perturbation methods have often been employed for bulk materials. These calculations typically involve a classification of the Feynman-type diagrams according to an expansion parameter 1/z, where *z* denotes the number of spins interacting with any given spin. Some of these non-bosonic techniques make use of the drone-fermion representation, which was originally quoted for spin $S=\frac{1}{2}$ operators by Mattis [19], to obtain a diagrammatic perturbation expansion. Subsequently, the drone-fermion method was generalized to higher spin values [20]. Calculations of the SW energy shift and damping with this technique have been presented for various bulk magnetic systems, including ferromagnets and antiferromagnets with Heisenberg exchange interactions only [21,22,23,24,25,26] and ferromagnetic materials with both exchange and dipole–dipole interactions [27]. An alternative diagrammatic method, that also employs a 1/z expansion, was proposed by Vaks et al. [28,29] and later expanded upon by others [30,31,32,33,34]. This latter technique, sometimes referred to as a spin projection method, and the drone-fermion method lead to results which are essentially equivalent.

In this work, we are concerned with the SW magnetic properties in the recently produced two-dimensional (2D) van der Waals ferromagnets (see, e.g., [35,36,37,38,39,40,41,42]). Remarkably, these materials are capable of existing in a monolayer form, with the magnetic ions occupying a 2D honeycomb lattice (analogous to that of graphene [43,44]). The stabilizing influence of the magnetic anisotropies (usually taken as single-ion anisotropy and/or Ising exchange anisotropy) allows for an out-of-plane spontaneous magnetization while maintaining consistency with the well-known Mermin–Wagner theorem [45]. The existence of SWs in the van der Waals ferromagnet CrI_3_ was established through Raman scattering and magnetic resonance experiments [46,47], verifying that there are indeed two branches of SW excitations, as expected, for a honeycomb lattice structure with two sublattices. Further Raman spectroscopy studies have been reported for other van der Waals ferromagnets, such as Cr_2_Ge_2_Te_6_ (see [48,49,50]), which are being explored for their potential applications in (e.g.) spintronics and magnetic memories.

Here, we specifically focus on effects for the renormalization of the SWs, particularly the SW damping, in monolayer van der Waals ferromagnets. In previous work by Mkhitaryan and Ke [51], the temperature-dependent renormalization of the magnetization factor and the SW energy were considered, but not the damping. In general, at low temperatures corresponding to the bosonic regime, it is known that the intrinsic processes in magnetic films that can give rise to SW damping occur due to three-magnon and/or four-magnon scattering (see [1,2,3,4,11,52,53,54]). The three-magnon processes may occur when dipolar interactions are present, leading to the “splitting” and “confluence” contributions. By contrast, the four-magnon scattering occurs even when the dipolar interactions are absent (or can be neglected). The 1/z expansion methods mentioned earlier, however, give an extra process for the intrinsic SW damping at higher temperatures that we shall call “spin disorder” damping. This occurs if an SW scatters off the instantaneous disorder due to a longitudinal spin component undergoing a large thermal fluctuation. Hence, this mechanism has some analogies with the so-called two-magnon damping discussed by McMichael et al. [55,56] and Arias and Mills [6]; this extrinsic mechanism occurs when a magnon scatters off static spatial inhomogeneities in a ferromagnetic sample. Therefore, the spin disorder damping in our work plays an important role only at higher temperatures typically above about $\frac{1}{2}T_{C}$, where the spin fluctuations are larger. Previously, the spin disorder damping mechanism has been successful in explaining experimental data, such as the neutron scattering linewidths for ferromagnetic EuO [57] and the asymmetrically broadened lineshapes for Raman scattering from antiferromagnetic FeF_2_ [58]. Other data for linewidths and lineshapes in magnetic materials have been reviewed in [7].

The structure of the paper is as follows. In Section 2, we outline the van der Waals (vdW) materials and the methodology employed. The theory is presented in terms of the drone-fermion perturbation technique and the 1/z expansion, adopting an appropriate form to calculate the SWs in a van der Waals ferromagnetic monolayer. Next, the Green’s function results giving the spectrum of non-interacting SWs are described. In Section 3, we show the results of calculating the self-energy diagrams that represent the higher-order SW renormalization effects (including the damping, in particular). The analytical and numerical estimates for the damping are then presented using parameters appropriate to Cr_2_Ge_2_Te_6_ as an example, and the overall discussion is given in Section 4.

## 2. Materials and Methods

### 2.1. Background Theory

**Figure 1:**
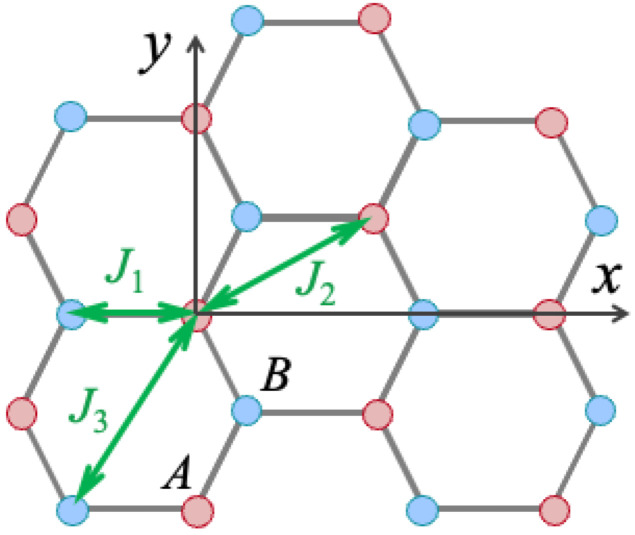
Geometry of the honeycomb lattice assumed for a van der Waals ferromagnet, showing the two types of sublattice sites (*A* and *B*) and the coordinate *x* and *y* axes. Examples of the nearest neighbor $J_{1}$, next-nearest neighbor $J_{2}$, and third-nearest neighbor $J_{3}$ exchange interactions are shown. The spins have their equilibrium orientation in the out-of-plane direction *z*.

A van der Waals ferromagnetic monolayer is modeled as a 2D honeycomb lattice of effective spins in the xy plane. There are two sublattice types of spin sites, denoted by *A* and *B* in Figure 1. An applied magnetic field $B_{0}$ acts along the perpendicular (*z*) axis, which is also the preferred direction of orientation for the spins. The spin Hamiltonian that is frequently adopted for a vdW ferromagnet [36,42] can be written as

(1) $$H=-\sum_{\langle m,n\rangle }J_{m,n}\left(S_{m}\cdot S_{n}+\sigma S_{m}^{z}S_{n}^{z}\right)-\sum_{m}D{\left(S_{m}^{z}\right)}^{2}-\sum_{m}bS_{m}^{z}\;,$$

where *m* and *n* denote sites on either sublattice and 〈m,n〉 in the first term indicates that the summations are taken over distinct pairs of sites. We include the exchange interactions $J_{1}$ to the three nearest neighbors on the opposite sublattice, interactions $J_{2}$ to the six next-nearest neighbors on the same sublattice, and interactions $J_{3}$ to the three third-nearest neighbors which are on the opposite sublattice. There are two possible contributions included in Equation (1) to the magnetic anisotropy, involving the Ising anisotropy with parameter σ and the single-ion anisotropy with parameter *D*. In general, both parameters are positive for the stability of the ferromagnetic ordering along *z*, but either one of them might be zero for a particular material. The final term in Equation (1) describes the Zeeman energy due to the applied field $B_{0}$, denoting $b=g\mu_{B}B_{0}$ where *g* is the Landé factor and $\mu_{B}$ is the Bohr magneton. In this work, we ignore, for simplicity, other possible interactions, such as dipole–dipole interactions or the antisymmetric Dzyaloshinski–Moriya exchange terms.

Next, we introduce the drone-fermion (DF) operators [19,21] by writing

(2) $$S_{m}^{+}=c_{m}^{†}\left(d_{m}+d_{m}^{†}\right),\;S_{m}^{-}=\left(d_{m}+d_{m}^{†}\right)c_{m},\;S_{m}^{z}=c_{m}^{†}c_{m}-\frac{1}{2},$$

where $S^{\pm }=S^{x}\pm iS^{y}$. Here, the fermion operator $c_{m}$ at site *m* satisfies the standard anticommutation relationships, and $d_{m}^{†}$ is an auxiliary (or “drone”) fermion operator that anticommutes with any of the *c* operators. The Hamiltonian in Equation (1) can be rewritten in terms of the DF operators, and the in-plane translational symmetry may be utilized to Fourier transform quantities from a site representation to a 2D wave vector $k=(k_{x},k_{y})$ representation. It is convenient to define Fourier transforms, denoted by v(k) and $v^{\prime }\left(k\right)$ for the inter- and intra-sublattice exchange terms, respectively, by

(3) $$\begin{array}{ccc} v(k) & = & J_{1}\left\{e^{-ik_{x}\;a}+2\;e^{ik_{x}\;a/2}\cos\left(\sqrt{3}\;k_{y}\;a/2\right)\right\}\\ & & +J_{3}\left\{e^{2ik_{x}\;a}+2\;e^{-ik_{x}\;a}\cos\left(\sqrt{3}\;k_{y}\;a\right)\right\}, \end{array}$$

(4) $$\begin{array}{ccc} v^{\prime }\left(k\right) & = & 2\;J_{2}\left\{\cos\left(\sqrt{3}\;k_{y}\;a\right)+2\;\cos\left(3k_{x}\;a/2\right)\cos\left(\sqrt{3}\;k_{y}\;a/2\right)\right\}, \end{array}$$

where *a* is the distance between spin sites along the side of any hexagon in Figure 1.

The Hamiltonian may now be expressed in two parts as $H=H_{0}+H_{1}$, where $H_{0}$ is quadratic in the operators and given by

(5) $$H_{0}=\left[b+b_{an}+S\left(1+\sigma \right)\left\{v\left(0\right)+v^{\prime }\left(0\right)\right\}\right]\sum_{k}\left(c_{Ak}^{†}c_{Ak}+c_{Bk}^{†}c_{Bk}\right).$$

Here, the subscripts *A* and *B* are associated with the operators to label the sublattice type. Also, $b_{an}$ is a single-ion anisotropy term, which takes the value (2S−1)D at low temperatures; it therefore correctly vanishes for a spin $S=\frac{1}{2}$ ferromagnet. The other part of the Hamiltonian is quartic in the operators, taking the form

(6) $$\begin{array}{ccc} H_{1} & = & -\frac{1}{2N}\sum_{k_{1},k_{2},q}\left\{\left(1+\sigma \right)v^{\prime }\left(q\right)\left(c_{Ak_{1}+q}^{†}c_{Ak_{2}-q}^{†}c_{Ak_{2}}c_{Ak_{1}}+c_{Bk_{1}+q}^{†}c_{Bk_{2}-q}^{†}c_{Bk_{2}}c_{Bk_{1}}\right)\right\}\\ & & -\frac{1}{2N}\sum_{k_{1},k_{2},q}\{\left(1+\sigma \right)v\left(q\right)\left(c_{Ak_{1}+q}^{†}c_{Bk_{2}-q}^{†}c_{Bk_{2}}c_{Ak_{1}}+H.c.\right\}\\ & & -\frac{1}{2N}\sum_{k_{1},k_{2},q}\frac{1}{2}v^{\prime }\left(q\right)\left\{\left(\phi_{Ak_{1}+q}^{†}c_{Ak_{2}-q}^{†}\phi_{Ak_{2}}c_{Ak_{1}}+\phi_{Bk_{1}+q}^{†}c_{Bk_{2}-q}^{†}\phi_{Bk_{2}}c_{Bk_{1}}\right)+H.c.\right\}\\ & & -\frac{1}{2N}\sum_{k_{1},k_{2},q}\frac{1}{2}\left\{v\left(q\right)\left(\phi_{Ak_{1}+q}^{†}c_{Bk_{2}-q}^{†}\phi_{Bk_{2}}c_{Ak_{1}}+\phi_{Bk_{1}+q}^{†}c_{Ak_{2}-q}^{†}\phi_{Ak_{2}}c_{Bk_{1}}\right)+H.c.\right\}, \end{array}$$

where H.c. is Hermitian conjugate and we denote the operator combination $d+d^{†}$ by ϕ. In order to study the spin correlations in this system, we next introduce the well-known imaginary time (or Matsubara) Green’s functions (see [59,60,61,62,63,64]) in a form appropriate for the DF technique by writing $C_{lk}\left(\tau \right)=\langle {\hat{T}}_{W}c_{lk}\left(\tau \right)c_{lk}^{†}\left(0\right)\rangle$ and $D_{lk}\left(\tau \right)=\langle {\hat{T}}_{W}\phi_{lk}\left(\tau \right)\phi_{lk}^{†}\left(0\right)\rangle$. Here, *l* is a sublattice label (*A* or *B*), the angular brackets denote an average taken using the Hamiltonian, and ${\hat{T}}_{W}$ is the Wick ordering operator. The transformation of the operators to the τ-representation is written, for example, as $c_{lk}\left(\tau \right)=e^{H\tau }c_{lk}e^{-H\tau }$. The Fourier components of these Green’s functions (GFs) in the frequency representation [59,64] will be denoted by $C_{lk}\left(i\alpha \right)$ and $D_{lk}\left(i\alpha \right)$, respectively, defining

(7) $$\begin{array}{c} C_{lk}\left(\tau \right)=-\frac{1}{\beta }\sum_{\alpha }e^{-i\alpha \tau }C_{lk}\left(i\alpha \right)\equiv -\frac{1}{\beta }\sum_{\alpha }e^{-i\alpha \tau }G\left(c_{lk};c_{lk}^{†}|i\alpha \right),\\ D_{lk}\left(\tau \right)=-\frac{1}{\beta }\sum_{\alpha }e^{-i\alpha \tau }D_{lk}\left(i\alpha \right)\equiv -\frac{1}{\beta }\sum_{\alpha }e^{-i\alpha \tau }G\left(\phi_{lk};\phi_{lk}^{†}|i\alpha \right), \end{array}$$

where α=(2m+1)π/β is a fermion frequency (*m* = any integer and $\beta =1/k_{B}T$). It is straightforward to show, following earlier DF calculations [20,27], that the “unperturbed” GFs evaluated with respect to $H_{0}$ are $C_{lk}^{0}\left(i\alpha \right)={\left\{i\alpha +b+b_{an}+S\left(1+\sigma \right)\left[v_{1}\left(0\right)+v_{2}\left(0\right)\right]\right\}}^{-1}$ and $D_{lk}^{0}\left(i\alpha \right)=2{\left(i\alpha \right)}^{-1}$.

At this stage, it is useful to comment on an important difference between the DF method and the low-temperature bosonic method mentioned earlier. Typically, the latter approach (see, e.g., [4,5,14]) involves a transformation to boson operators using the Holstein–Primakoff representation. Then, the square root of an operator expression is simplified by using a binomial expansion, which is terminated in an approximation where products of operators higher than quartic are ignored. This is well justified when $T\ll T_{C}$, but breaks down at higher temperatures. An advantage of the DF method is that it avoids any truncation.

**Figure 2:**
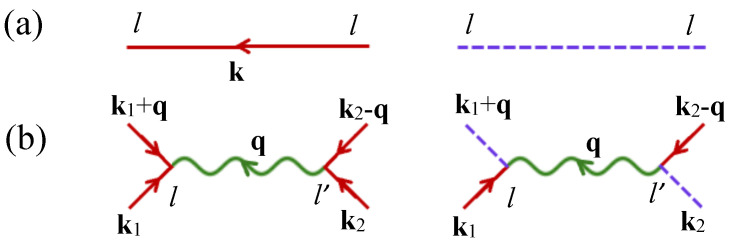
Diagrammatic representation used in the DF method: (**a**) the solid and dashed GF lines, $C_{lk}^{0}\left(i\alpha \right)$ and $D_{lk}^{0}\left(i\alpha \right)$, respectively, and (**b**) the generalized longitudinal and transverse interaction vertices involving v(q) or $v^{\prime }\left(q\right)$ according to the sublattice type at each end of the interaction.

The next stage in the DF method is to introduce a diagrammatic representation in which the GFs $C_{lk}^{0}\left(i\alpha \right)$ and $D_{lk}^{0}\left(i\alpha \right)$ are drawn as solid and dashed lines as in Figure 2, respectively, and there are two types of interaction vertices for $H_{1}$. The GF contributions can then be selected using the 1/z expansion parameter mentioned in Section 1. It is standard to incorporate a zeroth-order ${\left(1/z\right)}^{0}$ renormalization of the defined GFs, a step that is formally equivalent to mean-field theory. The spin fluctuations, giving the SWs and their interactions, will correspond to successively higher orders. By analogy with [20,27], the GFs are found (after incorporating all bubble-type or single-loop diagrams) to be $C_{lk}^{0}\left(i\alpha \right)={\left(\gamma +i\alpha \right)}^{-1}$ and $D_{lk}^{0}\left(i\alpha \right)=2{\left(i\alpha \right)}^{-1}$, where the effective mean-field quantity γ is

(8) $$\gamma =b+b_{an}+R_{0}\left(1+\sigma \right)\left[v\left(0\right)+v^{\prime }\left(0\right)\right]$$

at each site. Here, $R_{0}$ denotes the mean-field spin average $\langle S_{m}^{z}\rangle$ at any site, independent of label *m* by symmetry. For example, we have the standard mean-field expressions $R_{0}=\frac{1}{2}\tanh\left(\beta \gamma /2\right)$ and $R_{0}=2\coth\left(2\beta \gamma \right)-\frac{1}{2}\coth\left(\beta \gamma /2\right)$ in the cases of spin S=1/2 and 3/2, respectively [65,66]. These spin values will be used in the specific applications to be made later.

Conventionally, the SWs and their renormalization can be studied from a consideration of the poles of GFs written as $\langle {\hat{T}}_{W}S_{k}^{-}\left(\tau \right)S_{k}^{+}\left(0\right)\rangle$. Specifically, we employ the frequency Fourier components defined by

(9) $$F_{k}\left(i\eta \right)\equiv G\left(S_{k}^{-};S_{k}^{+}\;|\;i\eta \right)\;=\;\sum_{q,q^{\prime }}G\left(\phi_{q^{\prime }}^{†}c_{q^{\prime }+k};c_{q+k}^{†}\phi_{q}\;|\;i\eta \right).$$

**Figure 3:**
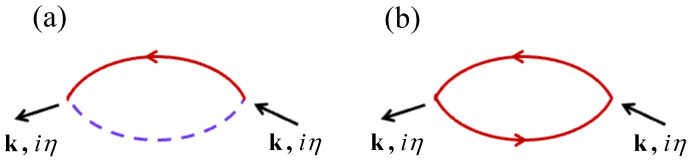
The single-loop diagrams contributing to the spin–spin GFs: (**a**) the transverse $F_{k}\left(i\eta \right)$ and (**b**) the longitudinal $L_{k}\left(i\eta \right)$.

**Figure 4:**
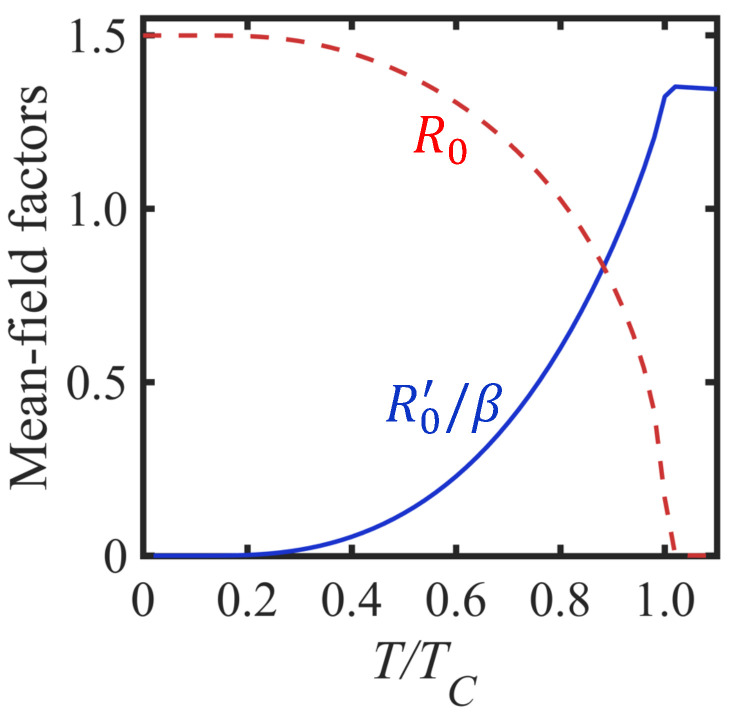
Plots for the temperature dependence of the dimensionless mean-field variables $R_{0}$ (dashed curve) and $R_{0}^{\prime }/\beta$ (solid curve), taking a small applied field such that $g\mu_{B}B_{0}/J=0.0001$. We assume parameter values for the S=3/2 ferromagnet Cr_2_Ge_2_Te_6_, as quoted in the text.

In this case, the label η=2mπ/β is a Matsubara boson frequency, since it involves sums or differences between fermion frequencies. A simple contribution to $F_{k}\left(i\eta \right)$ is just a single transverse loop diagram as depicted in Figure 3a. Its evaluation, using the standard diagrammatic rules (see [20]) and our results for $C_{lk}^{0}\left(i\alpha \right)$ and $D_{lk}^{0}\left(i\alpha \right)$, leads to $2R_{0}/\left(\gamma -i\eta \right)$ for both sublattices. Correspondingly, the single longitudinal loop in Figure 3b, which behaves like a longitudinal susceptibility, gives $\left(R_{0}^{\prime }/\beta \right)\;\delta_{i\eta ,0}$ by analogy with [20]), where $R_{0}^{\prime }=\partial R_{0}/\partial \gamma$ and $\beta =1/k_{B}T$ as before. It is easy to show that $R_{0}^{\prime }$, which characterizes longitudinal spin fluctuations, is negligibly small at low temperatures (behaving in this region like $e^{-T_{C}/T})$ but becomes of significance for any temperature *T* greater than about $0.5T_{C}$. This mean-field behavior is illustrated in Figure 4 for the van der Waals ferromagnet Cr_2_Ge_2_Te_6_, which has spin S=3/2. We assume approximate parameter values consistent with those given in [51]; specifically, we take $J_{1}=2.01$ meV, $J_{2}/J_{1}=0.08$, $J_{3}/J_{1}=-0.04$, $D/J_{1}=0.11$, and σ=0. For this material with its three different exchange constants, we have z=12, so the convergence in a 1/z expansion is good.

### 2.2. Spin Waves in Lowest Order

**Figure 5:**
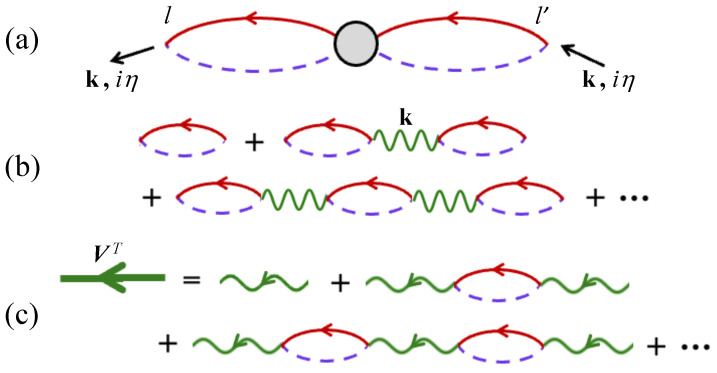
Renormalization of the transverse GF $F_{k}\left(i\eta \right)$: (**a**) the general form of any diagrammatic contribution; (**b**) the chain diagrams of single loops and interactions giving $F_{k}^{0}(i\eta$); and (**c**) the effective chain interaction $V^{T}\left(k,i\eta \right)$ in the transverse case.

Going beyond the single-loop approximation, the general form of any contribution to the transverse GF $F_{k}\left(i\eta \right)$ is shown in Figure 5a, where the shaded region schematically represents any allowed combination of single-particle GF lines together with the exchange interaction vertices. The specific combinations can be selected in accordance with the 1/z classification scheme. As in the earlier DF studies already cited, the lowest-order diagrams are those that have no internal wave vector label in a vertex, recalling that the k label introduced in Equation (9) is a fixed external label: the required contribution thus consists of a series of chain diagrams formed from single loops and interactions. The connecting exchange interactions (wavy lines) can either be of the type v(k) or $v^{\prime }\left(k\right)$, depending on the sublattice labels involved in the loops.

To accomplish the evaluation, it is useful to introduce 2×2 matrices (according to sublattice type) for single transverse loops and for single exchange interactions, denoting

(10) $$F_{k}^{loop}\left(i\eta \right)=\frac{2R_{0}}{\beta (\gamma -i\eta )}\left(\begin{array}{cc} 1 & 0\\ 0 & 1 \end{array}\right),\;\;v^{T}\left(k\right)=\frac{1}{2}\beta \left(\begin{array}{cc} v^{\prime }\left(k\right) & v(k)\\ v^{*}\left(k\right) & v^{\prime }\left(k\right) \end{array}\right).$$

We then need to sum a geometric series that arises from Figure 5b, which sums to give $F^{loop}{\left\{I-v^{T}F^{loop}\right\}}^{-1}\equiv F^{0}$, where I is the unit 2×2 matrix. This leads to the result

(11) $$\begin{array}{ccc} F_{k}^{0}\left(i\eta \right) & = & \frac{2R_{0}}{\beta \left(E_{1k}-i\eta \right)\left(E_{2k}-i\eta \right)}\left(\begin{array}{cc} \gamma -v^{\prime }\left(k\right)R_{0}-i\eta & v\left(k\right)R_{0}\\ v^{*}\left(k\right)R_{0} & \gamma -v^{\prime }\left(k\right)R_{0}-i\eta \end{array}\right). \end{array}$$

The above GF has simple poles at $i\eta =E_{1k}$ and $E_{2k}$, where we define

(12) $$\begin{array}{ccc} E_{1k} & = & \gamma -v^{\prime }\left(k\right)R_{0}-\left|v\left(k\right)\right|R_{0}\;(\mathrm{acoustic}\;\mathrm{SW}),\\ E_{2k} & = & \gamma -v^{\prime }\left(k\right)R_{0}+\left|v\left(k\right)\right|R_{0}\;\;(\mathrm{optic}\;\mathrm{SW}). \end{array}$$

These are recognized as being just the linear SW dispersion relations for a vdW ferromagnet, where we predict the existence of the two branches as seen, for example, by Raman scattering [46] from the magnons in CrI_3_. At low temperatures (on putting $R_{0}\to S$), the expressions are consistent with previous theoretical work (e.g., [51,67]).

It is convenient also to calculate a related quantity representing an effective chain interaction for the transverse exchange terms. This matrix quantity, which is denoted by $V^{T}\left(k,i\eta \right)$ and represented diagrammatically by a thick green line, is defined by the series of diagrams in Figure 5c. It is given algebraically by $V^{T}={\left\{I-v^{T}F^{loop}\right\}}^{-1}v^{T}$. Its matrix elements, which involve the same SW poles as before, are easily found to be

(13) $$\begin{array}{ccc} V_{11}^{T}\left(k,i\eta \right) & = & V_{22}^{T}\left(k,i\eta \right)=\frac{\beta \left(\gamma -i\eta \right)\{v^{\prime }\left(k\right)\left[\gamma -v^{\prime }\left(k\right)R_{0}-i\eta \right]+|v\left(k\right){|}^{2}R_{0}\}}{2\left(E_{1k}-i\eta \right)\left(E_{2k}-i\eta \right)}, \end{array}$$

(14) $$\begin{array}{ccc} V_{12}^{T}\left(k,i\eta \right) & = & {\left\{V_{21}^{T}\left(k,i\eta \right)\right\}}^{*}=\frac{\beta \left(\gamma -i\eta \right)\{v\left(k\right)\left[\gamma -v^{\prime }\left(k\right)R_{0}-i\eta \right]+v\left(k\right)v^{\prime }\left(k\right)R_{0}\}}{2\left(E_{1k}-i\eta \right)\left(E_{2k}-i\eta \right)}. \end{array}$$

**Figure 6:**
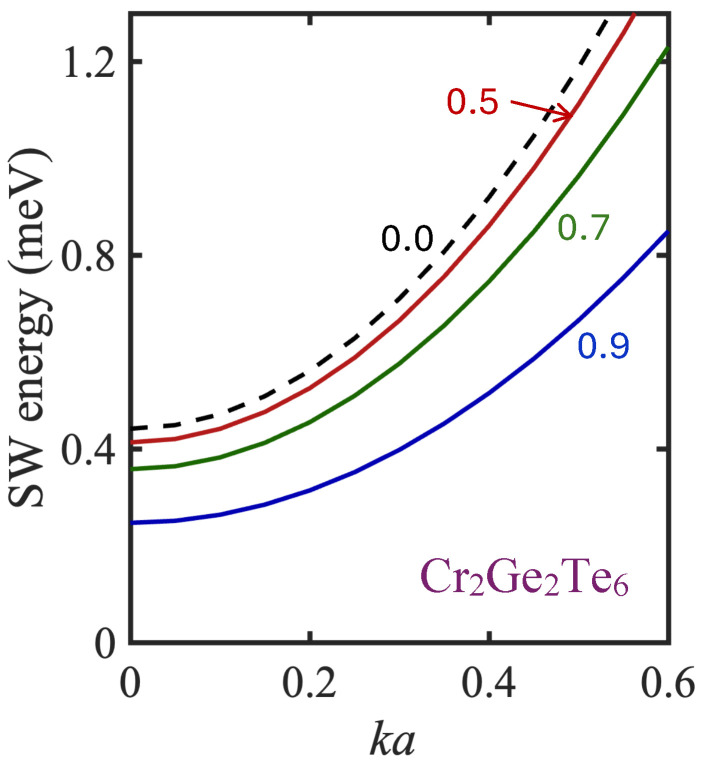
The calculated SW energies plotted versus ka for the acoustic (lower) branch in Cr_2_Ge_2_Te_6_ at small wave vectors and for several different temperatures below $T_{C}$. The curves are labeled with reduced temperature $\tau =T/T_{C}$ and the other parameters are given in the text.

The calculated SW dispersion (for $E_{1k}$ versus ka at a small magnitude of the wave vector) in the case of the lower (acoustic) branch is shown in Figure 6 using parameters for Cr_2_Ge_2_Te_6_. For comparison, the average Brillouin zone boundary wave vector corresponds to ka∼2.2. In this small wave vector regime, we have an approximate quadratic dependence on ka given by

(15) $$\begin{array}{ccc} E_{1k} & = & E_{0}+\left(3/4\right)R_{0}\left[J_{1}+6J_{2}+4J_{3}\right]{\left(ka\right)}^{2}, \end{array}$$

where the the SW energy gap is $E_{0}=b+b_{an}+3R_{0}\sigma \left(J_{1}+2J_{2}+J_{3}\right)$. The effective anisotropy field $b_{an}$ decreases with temperature, often with the assumption of a power law dependence, where $b_{an}\propto {\left(R_{0}/S\right)}^{n}$ and index n∼2 [68,69,70]. The behavior of $E_{1k}$ at zero temperature and at several elevated temperatures is illustrated in this figure.

Finally, the corresponding effective chain interaction for the *longitudinal* exchange terms is defined by $V^{L}={\left\{I-v^{L}L^{loop}\right\}}^{-1}v^{L}$, where

(16) $$L_{k}^{loop}\left(i\eta \right)=\frac{R_{0}^{\prime }}{\beta }\;\delta_{i\eta ,0}\left(\begin{array}{cc} 1 & 0\\ 0 & 1 \end{array}\right),\;v^{L}\left(k\right)=\left(1+\sigma \right)\beta \left(\begin{array}{cc} v^{\prime }\left(k\right) & v(k)\\ v^{*}\left(k\right) & v^{\prime }\left(k\right) \end{array}\right).$$

The matrix elements of $V^{L}\left(k,i\eta \right)$, which will be represented diagrammatically by a dashed thick green line, are straightforwardly obtained from the above definitions and are given by

(17) $$\begin{array}{ccc} V_{11}^{L}\left(k,i\eta \right) & = & V_{22}^{L}\left(k,i\eta \right)=\beta \left(1+\sigma \right)v^{\prime }\left(k\right)+\delta_{\eta ,0}R_{0}^{\prime }\beta {\left(1+\sigma \right)}^{2}\frac{H_{11}\left(k\right)}{H(k)}, \end{array}$$

(18) $$\begin{array}{ccc} V_{12}^{L}\left(k,i\eta \right) & = & {\left[V_{21}^{L}\left(k,i\eta \right)\right]}^{*}=\beta \left(1+\sigma \right)v\left(k\right)+\delta_{\eta ,0}R_{0}^{\prime }\beta {\left(1+\sigma \right)}^{2}\frac{H_{12}\left(k\right)}{H(k)}, \end{array}$$

where we denote

(19) $$\begin{array}{ccc} H_{11}\left(k\right) & = & v^{\prime 2}\left(k\right)+{\left|v\left(k\right)\right|}^{2}+R_{0}^{\prime }\left(1+\sigma \right)v^{\prime }{\left(k\right)[|v\left(k\right)|}^{2}-v^{\prime 2}\left(k\right)], \end{array}$$

(20) $$\begin{array}{ccc} H_{12}\left(k\right) & = & 2v^{\prime }\left(k\right)v\left(k\right)-R_{0}^{\prime }{\left(1+\sigma \right)v\left(k\right)[|v\left(k\right)|}^{2}-v^{\prime 2}\left(k\right)], \end{array}$$

(21) $$\begin{array}{ccc} H(k) & = & {\left[1-R_{0}^{\prime }\left(1+\sigma \right)v^{\prime }\left(k\right)\right]}^{2}-[R_{0}^{\prime }{\left(1+\sigma \right)|v\left(k\right)|]}^{2}. \end{array}$$

## 3. Results

### 3.1. Inclusion of Spin Wave Interactions

Now, to fulfill our objective of studying the SW properties at an elevated temperature (particularly above $0.5T_{C}$), we proceed to find the relationship connecting the SW renormalized energy and damping to the GF self-energy contributions that occur in higher orders of the 1/z expansion. Then, the specific choice of the self-energy terms will be given afterwards, and followed by their evaluation to deduce the SW damping results.

Proceeding by analogy with DF calculations applied to the other systems mentioned earlier, such as bulk 3D Heisenberg ferromagnets and antiferromagnets and some dipolar ferromagnets [20,22,23,27,71], we consider the renormalization of the spin–spin GF matrix defined diagrammatically in Figure 5b and given in lowest order (the single-loop approximation) by $F_{k}^{loop}\left(i\eta \right)$ in Equation (10). The required renormalization can be achieved formally by replacing the matrix $F_{k}^{loop}\left(i\eta \right)$ by the quantity $\left\{F_{k}^{loop}\left(i\eta \right)+\Sigma \left(k,i\eta \right)\right\}$, where Σ(k,iη) is a 2×2 matrix proper self-energy that contains the higher-order effects. It follows from this result that the modified poles of the renormalized GF will correspond to the determinantal condition that

(22) $$\det\left[I-v^{T}\left(k\right)\left\{F_{k}^{loop}\left(i\eta \right)+\Sigma \left(k,i\eta \right)\right\}\right]=0.$$

When the above 2×2 determinant is multiplied out, we obtain some terms that are independent of Σ, plus other terms that are linear and quadratic in the matrix elements of Σ. Since Σ is a small quantity in our perturbation approach, we neglect the quadratic terms. Retaining just the linear effects, we obtain a condition from Equation (22) that may be expressed as $\left(E_{1k}-i\eta \right)\left(E_{2k}-i\eta \right)-\Lambda \left(i\eta \right)=0$, where the self-energy term is $\Lambda \left(i\eta \right)=\beta \left(\gamma -i\eta \right)\left\{\left[v^{\prime }\left(k\right)\left(\gamma -v^{\prime }\left(k\right)R_{0}-i\eta \right)+{\left|v\left(k\right)\right|}^{2}R_{0}\right]\Sigma_{11}\left(k,i\eta \right)+\frac{1}{2}\left(\gamma -i\eta \right)\left[v^{\prime *}\left(k\right)\Sigma_{12}\left(k,i\eta \right)+H.c.\right]\right\}$. The next step is to make an analytic continuation for the complex boson frequency such that $i\eta \to E_{jk}+\left(\Delta E_{jk}-i\Gamma_{jk}\right)$, where $E_{jk}$ (with j=1,2) is one of the “unperturbed” SW dispersion solutions in Equation (12). Here, the real term $\Delta E_{jk}$ and the imaginary term $\Gamma_{jk}$ represent the SW energy correction and the damping, respectively, for the SW branch *j*. By rearranging the determinantal condition quoted above, along with using Equation (12) for the unrenormalized SW energies, we may conclude that an SW pole at $E_{jk}$ is shifted (or renormalized) to have the approximate value $E_{jk}+\left\{\Delta E_{jk}\left(k,E_{jk}-i0^{+}\right)-i\Gamma_{jk}\left(k,E_{jk}-i0^{+}\right)\right\}$, where for general iη, we find

(23) $$\begin{array}{ccc} \Delta E_{jk}\left(k,i\eta \right)-i\Gamma_{jk}\left(k,i\eta \right) & = & \frac{\beta (\gamma -i\eta )}{4|v\left(k\right)|R_{0}}\{[2v^{\prime }\left(k\right)\left(\gamma -v^{\prime }\left(k\right)R_{0}-i\eta \right)\\ & & +{\left|v\left(k\right)\right|}^{2}R_{0}]\Sigma_{11}\left(k,i\eta \right)+\left(\gamma -i\eta \right)\left[v^{\prime *}\left(k\right)\Sigma_{12}\left(k,i\eta \right)+H.c.\right]\}. \end{array}$$

In obtaining the above expressions, we have used the symmetry relations $\Sigma_{11}=\Sigma_{22}$ and $\Sigma_{21}=\Sigma_{12}^{*}$ for the ferromagnetic van der Waals monolayer. Also, we have approximated by evaluating the self-energies at the unrenormalized SW energy $E_{jk}-i0^{+}$ instead of self-consistently at the renormalized $E_{jk}+\Delta E_{jk}-i\Gamma_{jk}$. This type of approximation is commonly made in many-body theory (see, e.g., [59,60,63]) and is referred to as an “on-resonance” approximation. It is justified, as discussed later, when the SW excitations are well-defined in the sense that a necessary validity condition is

(24) $$\begin{array}{ccc} |\Delta E_{jk}-i\Gamma_{jk}| & \ll & E_{jk}. \end{array}$$

**Figure 7:**
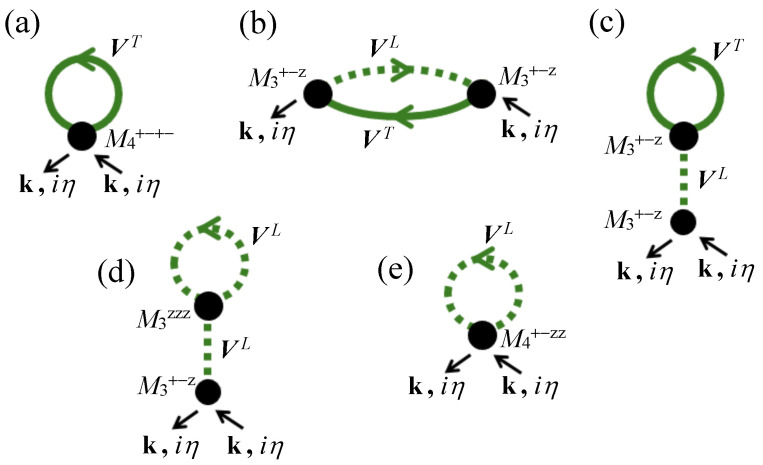
The leading-order diagrammatic contributions to the self-energy Σ(k,iη) as employed in calculating the SW energy renormalization and damping. Each diagram has labels k and iη for the incoming and outgoing wave vector and boson frequency, respectively. The generalized interaction chains $V^{T}$ and $V^{L}$ (solid and dashed green lines) were defined in Section 2, and the black circles are the interaction vertices.

As in the works cited earlier that employed the 1/z expansion, the diagrammatic contributions to the self-energies will be those that explicitly involve one extra wave vector (an internal wave vector which we label as q) in a summation for a diagram. The diagrams represent virtual processes in which either a transverse spin fluctuation (a spin wave) or a longitudinal spin fluctuation is emitted and subsequently adsorbed. After considering all possibilities, the resulting diagrams for Σ(k,iη) are those shown in Figure 7. We see that all these diagrams have the external labels for wave vector k and boson frequency iη entering and leaving. Also, they have the internal labels q and $i\eta^{\prime }$ to be summed over. They contain matrix elements of the generalized interaction chains $V^{T}$ and $V^{L}$ defined earlier; these chains are denoted, respectively, by the solid and dashed heavy green lines. The filled black circles $M_{n}$ represent interaction vertices (or junction points) connecting *n* other lines (n≥3); they have a general form represented as a closed ring with *n* one-particle GF lines and vertex points that may correspond to $S^{+}$, $S^{-}$, or $S^{z}$. From a formal perspective, they are just the analogs of the so-called “semi-invariants” described by Stinchcombe et al. [72] and the “vertices” of Vaks et al. [28]. Here, we are adopting the standard notation for $M_{n}$ employed in other DF calculations (see, e.g., [20], where their expressions are given in full).

The diagram that has the simplest form is that shown in Figure 7a. Physically, it describes a scattering process in which there is an incoming SW labeled with its wave vector and frequency. This SW interacts through a virtual process with another thermally excited SW (the solid green line in the diagram) which is subsequently reabsorbed to yield the outgoing SW. The relevant interaction vertex (the black circle labeled as $M_{4}^{+-+-}$) represents the probability for this process. The next diagram in Figure 7b is an example of a two-stage process in which there is a splitting of the incoming SW into another SW (solid green line) and longitudinal spin excitation (dashed green line). Again, these excitations subsequently recombine to yield the outgoing SW, but in this case there are two black circles for the interaction vertices (both labeled as $M_{3}^{+-z}$). The remaining three diagrams in Figure 7 have a similar interpretation and represent the other topologically allowed one-loop scattering processes that involve solid and/or dashed green lines. The total expression for the self-energy matrix Σ(k,iη) is found by summing the contributions from the individual diagrams shown in Figure 7. The total diagonal $\Sigma_{11}$ term is given by

(25) $$\begin{array}{ccc} \Sigma_{11}\left(k,i\eta \right) & = & \sum_{q,i\eta^{\prime }}[M_{4}^{+-+-}\left(i\eta ,i\eta ,i\eta^{\prime },i\eta^{\prime }\right)\;V_{11}^{T}\left(q,i\eta^{\prime }\right)+M_{4}^{+-zz}\left(i\eta ,i\eta ,i\eta^{\prime },i\eta^{\prime }\right)\;V_{11}^{L}\left(q,i\eta^{\prime }\right)\\ & & +M_{3}^{+-z}\left(i\eta ,i\eta^{\prime },i\eta^{\prime }-i\eta \right)M_{3}^{+-z}\left(i\eta^{\prime },i\eta ,i\eta -i\eta^{\prime }\right)\;V_{11}^{T}\left(q,i\eta^{\prime }\right)V_{11}^{L}\left(q-k,i\eta^{\prime }-i\eta \right)\\ & & +M_{3}^{+-z}\left(i\eta ,i\eta ,0\right)\left\{J_{11}^{L}\left(0,0\right)+J_{12}^{L}\left(0,0\right)\right\}\{M_{3}^{+-z}\left(i\eta^{\prime },i\eta^{\prime },0\right)\;J_{11}^{T}\left(q,i\eta^{\prime }\right)\\ & & +M_{3}^{zzz}\left(i\eta^{\prime },i\eta^{\prime },0\right)\;J_{11}^{L}\left(q,i\eta^{\prime }\right)\}], \end{array}$$

while the off-diagonal $\Sigma_{12}$ term, which has a contribution arising only from diagram (b) in Figure 7, is

(26) $$\begin{array}{ccc} \Sigma_{12}\left(k,i\eta \right) & = & \sum_{q,i\eta^{\prime }}M_{3}^{+-z}\left(i\eta ,i\eta^{\prime },i\eta^{\prime }-i\eta \right)M_{3}^{+-z}\left(i\eta^{\prime },i\eta ,i\eta -i\eta^{\prime }\right)\;V_{12}^{T}\left(q,i\eta^{\prime }\right)V_{21}^{L}\left(q-k,i\eta^{\prime }-i\eta \right). \end{array}$$

We next outline the process for summing over the internal boson frequency $i\eta^{\prime }$. This can be illustrated using diagram (a) in Figure 7, which is just the term proportional to $M_{4}^{+-+-}$ in Equation (25), but the method is similar for the other diagrams. On substituting for this vertex function (see [20]) and for its $V_{11}^{T}\left(q,i\eta^{\prime }\right)$ factor using Equation (13), we find

(27) $$\begin{array}{ccc} \Sigma_{11}^{\left(a\right)}\left(k,i\eta \right) & = & \frac{2}{\beta^{2}\left(\gamma -i\eta \right)}\sum_{q,i\eta^{\prime }}\left\{\frac{-R_{0}\left(2\gamma -i\eta -i\eta^{\prime }\right)}{\left(\gamma -i\eta \right)\left(\gamma -i\eta^{\prime }\right)}+R_{0}^{\prime }\left[1+\delta_{\eta -\eta^{\prime },0}\right]\right\}\\ & & \times \frac{\left\{v^{\prime }\left(q\right)\left[\gamma -v^{\prime }\left(q\right)R_{0}-i\eta^{\prime }\right]+|v\left(q\right){|}^{2}R_{0}\right\}}{\left(E_{1q}-i\eta^{\prime }\right)\left(E_{2q}-i\eta^{\prime }\right)}. \end{array}$$

Two cases now arise while carrying out the summation over $i\eta^{\prime }$. One of these comes from doing the contour integration in the complex frequency plane, making use of the residue theorem to take account of the poles (for $i\eta^{\prime }$) at the SW energies $E_{1q}$ and $E_{2q}$ and at the effective mean field quantity γ. Employing standard many-body theory techniques (e.g., see [59,60,63,64]), we obtain

(28) $$\frac{-\beta }{2R_{0}}\sum_{q}\sum_{j=1}^{2}\frac{{\left(-1\right)}^{j}\Phi \left(E_{jq}\right)}{\left|v\left(q\right)\right|\left(\gamma -E_{jq}\right)}n\left(E_{jq}\right),$$

where $n\left(x\right)\equiv {\left\{\exp\left(\beta x\right)-1\right\}}^{-1}$ defines the Bose–Einstein thermal factor for any *x*, *j*(=1,2) is an SW branch label, and the weighting factor Φ is

(29) $$\Phi \left(x\right)=\frac{2}{\beta^{2}\left(\gamma -i\eta \right)}\left\{R_{0}^{\prime }-\frac{R_{0}\left(2\gamma -i\eta -x\right)}{\left(\gamma -i\eta )(\gamma -x\right)}\right\}\{v^{\prime }\left(q\right)\left[\gamma -v^{\prime }\left(q\right)R_{0}-x\right]+|v\left(q\right){|}^{2}R_{0}\}.$$

An important conclusion reached using Equations (28) and (29), together with Equation (23), is that the above contribution made to the analytically continued renormalization term $\Delta E_{jk}\left(k,E_{jk}-i0^{+}\right)-i\Gamma_{jk}\left(k,E_{jk}-i0^{+}\right)$ is *real* rather than complex, and hence it gives no contribution to the SW damping in this order of perturbation.

By contrast, the other kind of contribution that arises due the $i\eta^{\prime }$ summation in Equation (27) comes from the presence of the factor $\delta_{\eta -\eta^{\prime },0}$. It results in a further contribution to the self energy $\Sigma_{11}^{\left(a\right)}\left(k,i\eta \right)$ of the form

(30) $$\begin{array}{c} \frac{2R_{0}^{\prime }}{\beta^{2}\left(\gamma -i\eta \right)}\sum_{q}\frac{\left\{v^{\prime }\left(q\right)\left[\gamma -v^{\prime }\left(q\right)R_{0}-i\eta \right]+|v\left(q\right){|}^{2}R_{0}\right\}}{\left(E_{1q}-i\eta \right)\left(E_{2q}-i\eta \right)}. \end{array}$$

**Figure 8:**
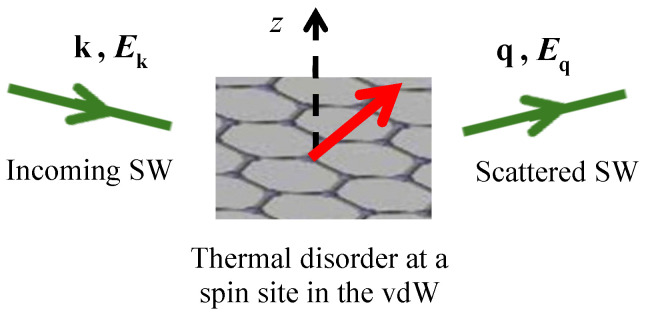
Schematic representation of the mechanism for spin disorder damping. There is an incoming SW with wave vector k and energy $E_{k}$, which scatters from a thermally disordered spin in the 2D lattice into another SW with wave vector q and energy $E_{q}$. The red arrow indicates a disordered spin vector participating in this process.

Now, when the analytic continuation $i\eta \to E_{jk}-i0^{+}$ is made, *both real and imaginary* parts will arise in the expression due to the denominators within the above q summation. These parts are obtained by using the formal identity

(31) $$\frac{1}{E_{jk}-E_{jq}-i0^{+}}=P\left(\frac{1}{E_{jk}-E_{jq}}\right)+i\pi \;\delta \left(E_{jk}-E_{jq}\right)$$

within the q summation, where P means that a principal value is taken. Hence, provided that the energy-conserving delta function $\delta (E_{jk}-E_{jq})$ can be satisfied for some wave vectors, there is now a contribution obtained for the SW damping. This two-magnon process is the spin disorder damping, which was mentioned earlier and is depicted schematically in Figure 8. We note that the contribution will be negligible in the low-temperature bosonic regime because of the associated factor $R_{0}^{\prime }$. As in the earlier DF calculations involving this mechanism, we anticipate that, at temperatures of about $0.5T_{C}$ and higher where spin deviations are large, it gives the dominant effect. The thermal spin fluctuations are larger at higher temperatures, where they represent a greater spin disorder in the system. Consequently, the scattering of the SWs is increased, reducing the SW lifetime and hence increasing the damping.

Using the above identity, the imaginary part of the analytically continued self-energy term in Equation (30) is

(32) ImΣ11(a)=πR0′β2(γ−iη)∑qj{v′(q)[γ−v′(q)R0−Ejk]+|v(q)|2R0}|v(k)|R0δ(Ejk−Ejq),

which may be substituted back into Equation (23) to obtain the contribution to the SW damping from that diagram.

The self-energy contributions from all the other diagrams in Figure 7 can similarly be evaluated. They come from the other terms in Equations (25) and (26), and typically they involve the same two types of contributions from the summations over the internal $i\eta^{\prime }$ label. Since we are interested in the SW damping, we may focus just on the imaginary parts of those self energies that give delta-function contributions analogous to that in Equation (32). There are, in fact, only two other such contributions: they are found to come from the diagonal $\Sigma_{11}^{\left(b\right)}$ and the off-diagonal $\Sigma_{12}^{\left(b\right)}$ terms for diagram (b) in Figure 7. The required expression obtained for the imaginary parts of these terms are

(33) ImΣ11(b)=πR0′β2R0(γ−iη)v(k)∑qjδ(Ejk−Ejq){v′(q)(γ−v′(q)R0−iη)+|v(q)|2R0}××[{v′(q−k)+(1+σ)R0′H11(q−k)H(q−k)}{−2R0R0′(γ−iη)+(R0′)2}+R02(γ−iη)2(1+σ)R0′H11(q−k)H(q−k)],

(34) ImΣ12(b)=πR0′β2R0(γ−iη)v(k)∑qjδ(Ejk−Ejq){v(q)(γ−v′(q)R0−iη)+v′(q)v(q)R0}××[{v′(q−k)+(1+σ)R0′H12(q−k)H(q−k)}{−2R0R0′(γ−iη)+(R0′)2}+R02(γ−iη)2(1+σ)R0′H12(q−k)H(q−k)].

### 3.2. Spin Disorder Damping Results

The important outcome from the previous section is that the damping for an SW at wave vector k on the branch *j* (=1,2), due to the spin disorder mechanism at higher temperature *T*, is given by an expression of the form

(35) $$\begin{array}{ccc} \Gamma_{jk}^{sd} & = & \pi R_{0}^{\prime }\sum_{q}\Psi_{j}\left(k,q\right)\;\delta \left(E_{jk}-E_{jq}\right), \end{array}$$

where Ψ is a weighting factor that depends on wave vectors k and q and on the SW branch number *j*, as well as on temperature and other parameters of our Hamiltonian (1). Before proceeding further, it is helpful to put this result into context with other possible damping contributions; the latter are those usually studied using bosonic methods and typically apply at lower temperatures than those considered here (see, e.g., [1,2,4,5,53]). These other contributions may occur through three-magnon and four-magnon processes, as we now describe briefly. The three-magnon damping becomes possible when the spin Hamiltonian contains magnetic dipole–dipole interactions; typically, it consists of terms with delta functions of the form $\delta (E_{k}-E_{q}-E_{k-q})$ and $\delta (E_{k}-E_{q}+E_{q-k})$, describing so-called “splitting” and “confluence” processes, respectively. Since our Hamiltonian in Equation (1) for a van der Waals monolayer does not involve dipole–dipole interactions, the three-magnon damping is absent in the current model. We note that these interactions could be included by modifying the renormalization methodology used in [71] for an ultrathin film with a simple-cubic lattice. The four-magnon damping is due to SW scattering processes in second order and involves delta functions of the form $\delta (E_{k}+E_{q}-E_{k+q^{\prime }}-E_{q-q^{\prime }})$ and similar. In principle, it can be calculated within the non-bosonic diagrammatic methods (as performed for bulk magnetic systems in, e.g., [24,29]), but it is a higher-order ${\left(1/z\right)}^{2}$ effect in the perturbation expansion. For this reason, and also because we are focusing on elevated temperatures, it will not be considered further here.

We now return to discuss our main SW damping result given in Equation (35). The expression for the weighting factor $\Psi_{j}\left(k,q\right)$ is found by substituting Equations (32)–(34) for the imaginary self energies into the renormalization Equation (23), taking $i\eta \to E_{jk}-i0^{+}$. The general result for the spin disorder damping is therefore obtained explicitly in our formalism, and the remaining wave vector summation for q over the 2D Brillouin zone could be accomplished numerically (e.g., as performed in analogous wave vector summations for the graphene lattice by following the procedure in [44,73]). This full analysis would be justified if experimental data (e.g., from measurements of the half-width of the SW peak in Raman and/or Brillouin light scattering) were to become available for a van der Waals ferromagnetic layer at suitable values of the wave vector k and temperature *T*.

### 3.3. Results for Cr_2_Ge_2_Te_6_

In the meantime, it is useful instead to examine some realistic special cases in which there are simplifications to the general results. Specifically, we shall focus on materials in which the intra-sublattice exchange (given by $J_{2}$) is small in magnitude compared to the combined effect of the inter-sublattice exchange (given by $J_{1}$ and $J_{3}$). This is the case, for example, in the van der Waals ferromagnets CrI_3_ and Cr_2_Ge_2_Te_6_. For the following numerical applications, we will again choose the latter of these materials, for which the relevant parameters (taken from [51]) were quoted earlier in Section 2.1. Thus, on approximating the expressions by taking $|v^{\prime }\left|\ll |v\right|$ for the exchange terms, we eventually find a simplified result for the damping of the lower SW branch (j=1), as given by

(36) $$\begin{array}{ccc} \Gamma_{1k}^{sd} & = & \frac{\pi R_{0}^{\prime }}{2\beta }\sum_{q}\frac{{\left|v\left(k\right)-\left(1+\sigma \right)v\left(q-k\right)\right|}^{2}}{1-R_{0}^{\prime }\left(1+\sigma \right)\left|v\left(q-k\right)\right|}\;\delta \left(E_{1k}-E_{1q}\right). \end{array}$$

Before discussing the numerical evaluation, it is worthwhile to point out some general features that can be deduced from this result in Equation (36). First, we note that, at any temperature below $T_{C}$, it yields a damping contribution that can become small provided that the reduced wave vector factor, ak, is sufficiently small. This is formally analogous to a result for the damping near $T_{C}$ in bulk ferromagnets (see [28,29]). Second, because the overall factor $R_{0}^{\prime }$ increases monotonically with *T* below $T_{C}$ (see Figure 4) and also the denominator term in Equation (36) decreases with temperature *T*, it follows that the predicted damping at any nonzero ka value increases with temperature. From Equation (24), there is a necessary validity condition to consider that requires $\Gamma_{1k}^{sd}\ll E_{1k}$. This may lead to a restriction on the range of the wave vector factor ak appropriate for any given temperature *T*.

At a relatively small magnitude *k* of the SW wave vector in the Brillouin zone (corresponding to the quadratic regime ka≲0.6 discussed earlier), we can make use of the property that the delta function in Equation (36) implies |q|=k. This leaves only an integration over a polar angle θ, representing the angle between the k and q vectors. We find eventually that Equation (36) can be rewritten in an integral form as

(37) $$\begin{array}{ccc} \Gamma_{1k}^{sd} & = & \frac{9\sqrt{3}R_{0}^{\prime }\left(J_{1}+4J_{3}\right)}{4\pi \beta R_{0}\left(1+\sigma \right)}\;\times \\ & & \int_{0}^{\pi }\frac{\left\{\left[\sigma +\frac{1}{4}a^{2}k^{2}\right]-k^{2}a^{2}\left(1+\sigma \right)\sin^{2}\left(\theta /2\right)\right]{\}}^{2}d\theta }{\left\{1-R_{0}^{\prime }\left(1+\sigma \right)3\left(J_{1}+J_{3}\right)\right\}+R_{0}^{\prime }\left(1+\sigma \right)3\left(J_{1}+4J_{3}\right)k^{2}a^{2}\sin^{2}\left(\theta /2\right)}. \end{array}$$

**Figure 9:**
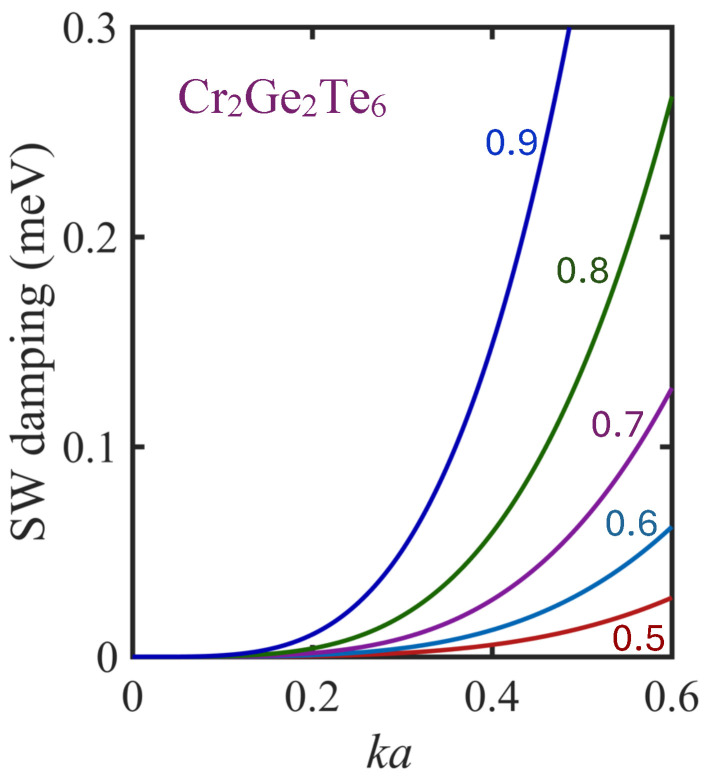
Estimates for the dominant SW damping $\Gamma_{1k}^{sd}$ due to the spin disorder scattering in a Cr_2_Ge_2_Te_6_ monolayer film, plotted versus the reduced in-plane wave vector ka for the several values of the reduced temperature $\tau =T/T_{C}$, as indicated. The results are for the lower SW branch 1 at a relatively small magnitude of ka in the Brillouin zone.

The remaining θ-integration, which ranges over all spatial directions from 0 to π, can be carried out using any standard numerical integration package. Here, we have employed a package for definite integrals available in MATLAB (version 9.13.0) [74], which has inbuilt criteria to ensure good accuracy (with relative errors being much less than 0.1 per cent in this case). We have employed values for the exchange factors, anisotropy, and applied magnetic field corresponding to a Cr_2_Ge_2_Te_6_ monolayer as quoted in Section 2.1 and the caption to Figure 4. Ranges of different values for ka (up to 0.6) and *T* (up to $0.9T_{C}$) were employed to find the results for the damping $\Gamma_{k,1}^{sd}$ shown in Figure 9. Here, we have plotted $\Gamma_{k,1}^{sd}$ (in meV units) against dimensionless wave vector ka for several different temperature values $\tau =T/T_{C}$. It is seen that the damping is predicted to increase sharply with respect to both ka and τ in the range considered. The wave vector dependence is approximately proportional to $k^{4}$ in the lower part of the range. The damping values predicted here (of order up to 0.2 meV) would correspond roughly to a Raman scattering peak with full width of about 3 cm^−1^ in wavenumber units. Although the Curie temperature for monolayer Cr_2_Ge_2_Te_6_ is variously quoted as being in the 40–60 K range (see [48,50]), it can be manipulated and increased to higher values with the application of lattice strain [50,75]. As an aside comment, we note that in addition to the ferromagnetic semiconductor phase for Cr_2_Ge_2_Te_6_ considered in our work, there can also be a metallic phase [76], for which the exchange parameters are different. The applicability of our present type of analysis to the metallic phase would depend on how the effective Hamiltonian for the system is changed compared to Equation (1).

**Figure 10:**
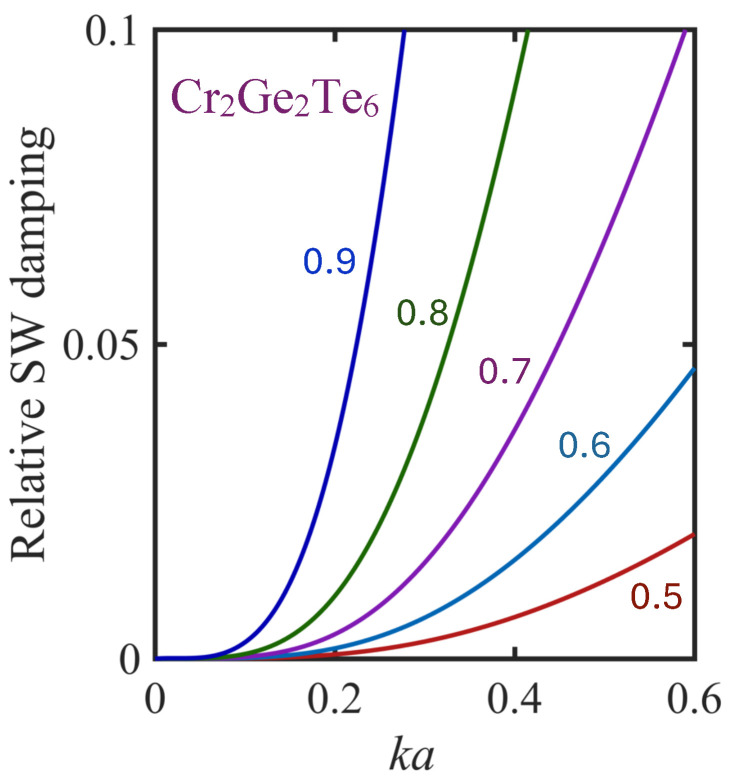
The same as in Figure 9 but for the relative damping $\Gamma_{1k}^{sd}/E_{1k}$ plotted versus ka at different temperatures.

It is important also to examine the behavior of $\Gamma_{1k}^{sd}/E_{1k}$, since we established earlier that this ratio should be small compared to unity as a validity condition for well-defined SW excitations. The results obtained for a Cr_2_Ge_2_Te_6_ monolayer film are shown in Figure 10. It is again the case that $\Gamma_{1k}^{sd}/E_{1k}$ increases monotonically with increasing ka and τ. We note that, for the curves with τ≤0.7, the validity condition can be convincingly satisfied in the range of ka plotted here. Thus, if we adopt the criterion, for example, that $\Gamma_{1k}^{sd}/E_{1k}≲0.1$, we see that at the higher temperatures with τ=0.8 and 0.9, the validity range for ka is reduced to being less than about 0.4 and 0.3, respectively.

## 4. Discussion

In conclusion, we have used a previously established non-bosonic diagram perturbation technique to investigate the behavior of the SW damping in 2D monolayers of ferromagnetic van der Waals materials. An advantage of this unconventional formalism is its validity for a wide temperature range below the Curie temperature $T_{C}$. In this regard, it avoids use of a Holstein–Primakoff-type transformation (where the operator expansion is truncated for $T\ll T_{C}$) to boson operators. The calculations were instead developed in terms of the DF diagrammatic representation and its generalizations [19,20,21,24,57]. Also, we point out that equivalent results could alternatively be derived from other techniques, such as the spin-projection method of Vaks et al. [28,29,31]. The references cited above were all for 3D magnetic systems, but we note that the DF method was recently employed in [71] to study the role of dipole–dipole interactions on the SW damping in ferromagnetic films of permalloy with cubic symmetry. By contrast, in our present work, we are considering a very different structure, namely the honeycomb structure of 2D vdW ferromagnets. These novel materials exhibit three different exchange constants (which compete with one another) and two interpenetrating sublattices (see Figure 1), giving technical differences, such as the use of the 2×2 matrix representation in Section 2, compared with [71] in the development of the theory. Also, the previous dipole–dipole terms are absent in this work, since they will usually be small compared to the other types of anisotropy that are included here and serve to stabilize the magnetic order in the 2D vdW materials. Overall, this has led to substantial differences here compared to [71].

The SW renormalization effects of energy shift and damping occur in higher orders of the perturbation technique. For the vdW monolayer, we evaluated the ${\left(1/z\right)}^{1}$ contributions, leading to the damping of the SWs at higher temperatures as a consequence of the spin disorder scattering mechanism. We note that, if we extend our analysis to the next order of perturbation, we would find the damping contribution due to four-magnon scattering processes (e.g., as in [1,2,3,4,77,78]) in the vdW system. By contrast, the spin disorder damping is a type of two-magnon scattering with a general dependence given by Equation (36). A magnon scatters off the thermal disorder in a *longitudinal* spin component, so it becomes important only at higher temperatures. Some examples of experimental work, where the spin disorder damping mechanism is evident in 3D materials, were given earlier.

As well as deriving the full formalism with general values of the three exchange terms taken into account, we made numerical applications here for the van der Waals ferromagnet Cr_2_Ge_2_Te_6_, utilizing the property of the intra-sublattice exchange term $J_{2}$ being small compared to the overall effect of the inter-sublattice exchange terms $J_{1}$ and $J_{3}$. Broadly similar results can be obtained for CrI_3_, for which the SWs have been studied by Raman scattering, but only at low temperatures below $T_{C}$, as mentioned earlier in the Introduction [46]. There is a similar temperature limitation for the optical spectroscopy studies applied to Cr_2_Ge_2_Te_6_. More generally, our theory would also be applicable to a wide range of transition-metal trihalides of the form MX_3_ with M = Cr or Ru and X = Cl, I or Br. We hope that our theoretical results developed here will stimulate measurements of the SW damping at elevated temperatures for this and similar materials, e.g., by data obtained for the line widths of the SW resonances in Raman scattering.

Some extensions to the present work that would be of interest include studying the SW renormalization and damping in vdW bilayers (e.g., formed from CrI_3_ or Cr_2_Ge_2_Te_6_ either with or without introducing a Moiré rotation) [41,79] and in vdW Néel-type antiferromagnetic monolayers (e.g., of MnPS_3_ or MnPSe_3_) [80]. We mentioned earlier the possibility of extending the formalism to include dipole–dipole interactions as an additional anisotropy that would allow three-magnon scattering. This could be of interest in materials where the other anisotropies (single-ion or Ising-type) are relatively small, as in the Mn compounds just mentioned. It would also be useful to make a systematic numerical study of the effects on the SW damping of varying the external applied magnetic field. Finally, a further possibility would be to include edge effects in a vdW monolayer (see, e.g., [81]).

## Data Availability

All of the data present in this paper will be made available upon reasonable request. Please contact the corresponding author for further information.
